# AI-powered creative stimulus: the ascent of virtual virtuoso entrepreneurship

**DOI:** 10.3389/frai.2025.1605855

**Published:** 2025-06-26

**Authors:** Ajimon George, Maria Susan Mathew

**Affiliations:** Department of Commerce, Marian College Kuttikkanam Autonomous, Kuttikkanam, India

**Keywords:** art, artificial intelligence, artrepreneurs, social media, entrepreneurship, artists, virtuoso

## 1 Introduction

The art world has undergone a revolutionary reshaping by introducing social media in the contemporary era. The traditional art world relied more on the bilateral relationship between artists and collectors, whereby established institutions were the bridge between the parties. Artists in conventional times relied solely on art galleries, which showcased and marketed their works through exhibitions and displays. Artists developed their crafts, worked with galleries and dealers, and participated in fairs and competitions to build relationships. They concentrated more on the sale of their artworks. As time passed by, the world of art came across a trend combining entrepreneurial skills and artistic talents. Here comes the evolution of art entrepreneurship, more commonly stated as artrepreneurship. Artrepreneur is resourceful and merges his creative ability and business acumen to establish a sustainable artist career. Artrepreneurship is the process of bringing an artist's creative capabilities into a physical form so that it can be commercialized widely (Hoffmann et al., 2021). Arts and Cultural Entrepreneurship encompasses several key elements that highlight the affiliation between the arts, culture, and entrepreneurial activities. Noonan (2021) coined the term “Creative Class”, which refers to a subset of the professional community which engages in creative vocations. The creative class is believed to enhance fiscal growth and community revitalization. Artrepreneurship itself is regarded as a segment of entrepreneurial activity that includes career management, the utilization of crowd funding platforms, and the adaptation to dynamic environments (Noonan, 2021). Social media has paved the way for disrupting traditional art marketing, fostering more accessibility and democracy in the creative community. The virtual world enabled the virtuosos, i.e., artists, to attain wider exposure and broader audience reach. However, the volume and magnitude of significant content on social media platforms act as a barrier for the artists to catch the eye. The importance of Artificial Intelligence (AI) here becomes apparent. Blending Artificial Intelligence (AI), social media, and entrepreneurship in the cultural and creative world has created an exciting and intricate new paradigm. AI has a transformative impact on innovation and creativity. AI redefined the criteria of artistry by re-evaluating the question of who can be considered an artist, which prompted a reconsideration of the capabilities, subjectivity, emotions, and creativity in art (Zhao and Zhang, 2023). The creative force and innovative design concept driven by AI enable the diverse expression of ideas and technologies that enhance the growth of art. A quantum leap from traditional thinking patterns and aesthetic sense is attained, enhancing the visual appeal and artistic value of creations (He, 2022). The evolving relationship between Artificial Intelligence and creative businesses explains that AI has the potential to be used as a collaborative assistant, which can be used to enhance creative productions rather than considering it as a replacement to human creativity. The integration of Artificial Intelligence is promising in nature which states that increased use of AI can lead to more content creation and artistic expression. The relationship explains the synergy between creativity and Artificial technologies in shaping future creative ventures (Anantrasirichai et al., 2025). This article examines how the integration of Artificial Intelligence and artrepreneurship reshapes the creative landscape. As artists entrepreneurs navigate the ever-evolving virtual environment, they face a unique set of opportunities and challenges that demand careful consideration, which is discussed briefly. It also contextualizes AI integrated art within existing creativity research and addresses the ethical concerns regarding the role of AI in creative productions.

## 2 Methodology

The article is an opinion piece, which employs a conceptual approach that explains the transformative impact of Artificial Intelligence on the artrepreneurship business. The study strictly relied on secondary data sources in discussing AI-powered virtual virtuoso entrepreneurship. A conceptual research approach is adopted in the study with existing theory and literature dependence to support the main claim, which is primarily interdisciplinary and focuses on perspectives from entrepreneurship, art and artificial intelligence. Desk research methodology is utilized in the study sourcing information from reputed journals and reputable online sources. Data sources include scholarly literature and research studies on AI integration in creative industries, digital entrepreneurship and social media marketing. It also provides case studies on AI-assisted artistic ventures and ethical considerations for employing AI-generated art. Also, a comparative analysis between traditional virtuoso entrepreneurship and AI-integrated virtuoso entrepreneurship is included, which sheds light on the evolving nature of artistic production and marketability in the era of Artificial Intelligence. As, an opinion-based commentary, the article is limited to be interpretative rather than empirical. The absence of primary data constrains the generalizability of the facts discussed.

### 2.1 Theoretical framework: AI and creativity research

M. Rogers', Diffusion of Innovation theory put forth a valuable framework for understanding the adoption of Artificial tools by art entrepreneurs. The theory underscores how a social system adopts and communicates innovations, particularly in a rapidly growing economy. In the perception of Rogers, diffusion is explained as a process by which novelty is communicated through a specific medium among the community. In a creative context, Artificial Intelligence enabled tools represent innovations that improve creativity, feasibility and market reach for art entrepreneurs (García-Avilés, 2020).

Guilford's Divergent Thinking Model involves four vital capabilities that are necessary for evaluating creative thinking in different tasks. These capabilities include fluency, flexibility, elaboration and originality. Divergent thinking is essential for the assessment of creative abilities as it involves multiple ideas or innovations. It can also enhance the process of content creation, assessment and collaboration eventually leading to high-quality creative outputs. Artificial Intelligence can replicate or enhance human creativity by exploiting different dimensions of the human thinking process (Forthmann et al., 2019).

Mihaly Csikszentmihalyi's systems approach offers Systems Model of Creativity which emphasizes creativity as an outcome of complex systems rather than individual efforts. This framework can enrich the domain of creativity by combining it with Artificial Intelligence. New dimensions of collaborations, power dynamics and personalization can be explored, ultimately leading to better utilization of the creative process in current scenario art practices (McIntyre, 2008).

### 2.2 Current state of artificial intelligence in virtuoso venture

The integration of AI and arts has been showing rapid evolution trends, significantly impacting ingenuity and value perceptions. AI can be employed in the creation of all types of art products, tangible or intangible, thereby enabling diversity in product types. The increasing trend of adoption of AI tools and human-AI collaborations in creative industries indicates how humans embrace AI in their cultural and creative ventures. Surprisingly, awareness of AI involvement in artistic content creation has negligible impact on the apparent value of the artworks. Art consumers are less bothered about machine involvement; even fully artificial artworks are demanded with significant value. Counterintuitively, the lack of human touch does not limit the emotional response of the art enthusiasts. Most AI-generated artwork does not fail to trigger the emotions of the art audience (Tigre Moura et al., 2023). In essence, AI art is poised to significantly reshape our understanding of creativity and human emotion in artistic contexts. The advent of AI-generated art is set to revolutionize our perception of flair, desire, self-consciousness, and emotional expression of art.

### 2.3 Prospects of artificial intelligence in virtuoso venture

The track of AI in art moves toward more individualized and hands-on exposure. The advancement of AI is also paving the way for the formation of novel art forms. AI uses its ability to utilize large databases, leading to the reflection of human sentiments and sensations, offering insights into complex patterns in the art world. The blend of aesthetics and data science provides shared and enhanced creativity. The collaboration of humans and AI opens the way for new possibilities of artistic endeavors and novelty. AI also acts as a tool to evolve and explore new ways of creativity (Wingström et al., 2024). The enhanced creativity enables the establishment of unique brand identity and marketing techniques that are rare and distinctive. New art products can be developed based on the personal tastes and experiences of the art customers. Art creation can be time-consuming and expensive. Artists also face art drainage at times. AI can aid art creation based on descriptions, enabling artrepreneurs to save considerably in costs and time. Furthermore, AI combines science and art. Artificial Intellect links computer science and creative abilities, offering new thoroughfare toward multidisciplinary collaboration (Wingström et al., 2024). Artrepreneurs need to test and refine their creative capabilities quickly. AI can be a powerful tool for rapid prototyping, where ideas can be converted into tangible forms from paper to digital. AI allows entrepreneurs to maximize the exhibition value, which enables entrepreneurs in the art industry to attract art audiences and increase the scope of market values and networks (Kalpokas, 2023). In their case study, Wallace et al. (2024) underscore the potential of AI as an instrument for extemporizing and improvising traditional dance and transforming abstract movements. The study suggests that deviating from realism can benefit creative practices positively, emphasizing the relevance of affiliation with specialists in evaluating and utilizing AI in artistic contexts.

### 2.4 Challenges of artificial intelligence in virtuoso venture

Artificial Intelligence, despite its opportunities, has numerous issues to be addressed. Usually, customers possess a negative attitude toward AI-generated creative ideas, probably due to a need for more education and awareness about AI and issues in their perceptions toward AI. Also, there is a fear of AI replacing human abilities as creative producers. Also, AI limits basic human instincts and emotions, which can restrict the wholesome creation of human interfered arts. AI art acts as a significant hazard to the traditional art market, where traditional artists are engaged in creating art based solely on their ideas and skills. Their human-inherited exertions go unnoticed by the rise of AI reliability (Kalpokas, 2023). Moreover, creating art based on queries and descriptions is not effortless, as AI may struggle to combine its cue conversion skills with creative composition (Wingström et al., 2024). Reconceptualization or redefinition of art is a possible threat that can impact human creativity. If AI-generated art can pass the “Turing test” by creating aesthetically pleasing artwork, a shift in understanding of creativity can occur. Discrepancies in artistic value and identity are another possible threat of AI-integrated art generation. The distinction between subjectivity and objectivity of art is another potential question regarding human involvement. AI-generated art is objective whereas traditional art is more subjective in nature. If art can be judged based on objective criteria through AI integration, AI can be programmed based on these standards. This can challenge human involvement, which is primarily based on emotions and living experiences, in creative endeavors (Hong and Curran, 2019). Entrepreneurs face data privacy and security issues. AI-generated art can also attract copyright issues, leading to complex issues relating to ownership, which leads to reluctance from the side of artist consumers. They also question authenticity and creativity concerns. Artrepreneurs struggle to attain confidence and market acceptability from the art community. The shift toward exhibition value impacts the perceptions of the artist community and the approaches toward acknowledgment of arts. This transition in the paradigm gives rise to the questioning of the intrinsic value of arts and their commerciality and marketability, which subsequently affects entrepreneurs in the field of art and creative areas (Kalpokas, 2023). Latikka et al. (2023) in their case study on attitudes of respondents toward AI Art, suggest the respondents exhibited a less positive attitude toward the integration of AI in the artistic community compared to other areas like healthcare or construction technology. Perception complexities suggest that AI art is strange, cold, or scary, reflecting a need for more familiarity and comfort with AI in creative endeavors. The case study reveals the complexity of attitudes toward AI integration in art, emphasizing the importance of psychological needs and contextual factors. The results suggest that while there is curiosity about AI's potential in art, there are also significant concerns that need to be addressed to foster acceptance.

### 2.5 Ethical and legal considerations of artificial intelligence in virtuoso ventures

The advent of AI has revolutionized the realm of art, reshaping the creative landscape and challenging traditional norms of authorship and creation. While the AI advancement offers numerous possibilities, it also raises a host of ethical and legal questions requiring careful consideration. The primary concern is the moral and legal implications of content created and uploaded by humans. Ethical concerns cover originality, authenticity, and creativity issues. Many artists raise doubts about the genuineness of original works. They argue that AI imitates existing ideas—the lack of self-awareness and emotional depth in a creative process leads to the authenticity of AI-generated works. Moreover, many artists debate whether AI can be truly creative, as creativity is a process that involves personal experiences and emotions, which AI lacks. It also touches on the aspect of motivation and intent behind AI-created art, questioning whether AI-generated art has the same intentions as those of human artists. Legal concerns revolve around ownership and the property rights of art. The ownership question is, in fact, a bit tricky—“Who is the real owner of the AI generated art Programmer, User or AI itself?”. This dilemma complicates the legal environment of AI-generated art. An additional critical area of legal focus is the Intellectual Property Rights landscape. Current laws may not be sufficient to address the challenges of AI art, which brings to attention how to protect both human artists and AI-generated art. The effect of AI-integrated art has implications for human artists, which questions the role of human creativity, subsequently leading to perplexities in future artistic professions (Liu, 2023). Moreover, people often have an aversion toward AI-generated art because of the need for familiarity and safety issues. There comes an obligation to educate the public about AI and machine intelligence to eradicate delusions and fears. There are concerns regarding user experience and autonomy. Likewise, cultural differences shape people's perceptions across societies, raising the ethical question of cultural sensitivity in AI-generated art, which is crucial for AI rollout (Latikka et al., 2023). To summarize, the ethical and legal aspects revolve around genuineness, security, impact on human creators, user freedom and Intellectual Property Rights. By exploring these ethical dilemmas, the human experience of art can be responsibly and morally enriched.

### 2.6 Traditional virtuoso entrepreneurship vs. AI integrated virtuoso entrepreneurship—comparison

The study comparison is given in Table 1, traditional virtuoso entrepreneurship vs. AI integrated virtuoso entrepreneurship—comparison.

**Table 1:** Traditional virtuoso entrepreneurship vs. AI integrated virtuoso entrepreneurship.

**Key issue**	**Traditional approach**	**AI- integrated approach**	**Examples of platforms that offer AI aid**
Creativity	Based on individual skills, life experiences, vision, enthusiasm, experimentation etc.	Inculcation of AI tools for idea generation from scratch and automating tasks	Using Prodia, Leap AI, Midjourney to generate ideas and rough sketches based on prompts.
Creation techniques	Manual art creation like painting, sculpting, music, dance, ceramics	Image generation, sound generation, animation, 3D modeling	Softwares like Stable diffusion for creation of hyper realistic images, MuseNet in creation of music, ZBrush for sculpting, Xsens for dance, Blender for Ceramic art can be used for intensifying artwork.
User engagement	Physical interactions, art galleries and exhibitions	Digital and virtual platforms, augmented reality, interactive platforms	Platforms like DALL- E 2, RunwayML, Roblox enhances user engagement.
Legal consequences	Traditional copyright laws and intellectual property rights.	New legal landscape surrounding AI-generated art which navigates through new territories.	Creation and trading of AI-generated NFTs (Non-Fungible Tokens) on Blockchain platforms.
Economic aspects	Conventional art markets, sale of arts, commission.	Social media marketing, AI-generated NFTs, patents of digital art.	An artist granting license of an AI generated art to other artists.
Ethical considerations	Concerns regarding authenticity and originality	Issues of bias, transparency and misuse of AI tools.	Non- disclosure of AI-integration in creative process

Source: Authors' own.

### 2.7 AI-powered virtual virtuoso entrepreneurship: examining the possibilities of AI integrated social media artrepreneurship

Arts and creative endeavors have been democratized in the era of social media to a large extent, but the introduction of AI in social media artrepreneurship is a paradigm shift. AI integration in art entrepreneurship is both transformative and disruptive. It holds huge promise as it can affect how art is created and how art can be marketed and sold.

AI acts as a muse in generating novel ideas, deciding the color palettes, deciding the pace of the music, converting recordings to music sheets, assisting choreography, rehearsal revolutions and what more. By collaborating with Artificial Intelligence and Arts, artists break the boundaries of human imagination. The unification of AI in social media could leverage the experiences of social media users, attracting more users toward the art community, and thereby enhancing deeper connections between artists and their audiences. AI aid can free artists from artist drainage. AI can also help in marketing strategies. Artists can rely more on their passion, converting their passion into a successful career. However, the use of AI could create art floods in the market, making it difficult for human artists to stand out and use their full potential. Ethical concerns and authenticity questions will definitely create pitfalls. It is better to keep a symbiotic link between human and machine interference. Artists must ensure a balance between AI assistance and artistic visions to capture the unique essence of artistic work. At the same time, AI can be used to focus more on business processing and leveraging social media marketability. The key is to exploit the merits of AI by not compromising the human touch. AI-integrated social media artrepreneurship is not about the replacement of artists by machines but about enfranchising them, fostering a new era of creative exposure and exploration. The prospect of AI-integrated social media artrepreneurship is both thrilling and volatile. Ultimately, the key to success lies in the ethical usage of artificial assistance, ensuring human elements, thereby fostering marketability and sales.

## 3 Discussion

Integrating AI into the sphere of creativity marks a revolution in artistic expression. Regardless of AI-integrated art's threats, it is becoming a powerful tool that can accelerate artistic abilities and open up new frontiers of creative capabilities. From generating concepts and ideas to enhancing overall art creation, technological innovation enables different experimentation and the creation of unimaginable artworks. However, as discussed earlier, the sophistication of AI raises concerns regarding authenticity, originality and human contribution to the creative community. Instead of mere substitution, artists should inculcate AI as a measure to leverage their creative endeavors. Moreover, AI models generate visual arts from internet datasets, which often reflect societal biases, stereotypes and imbalances. These biases can be reflected in AI generated outputs. This is, rather a consequence of trained data and lack of oversight in curation. AI generated art is not immune to such biases often leading to historical disparity, ethnic stereotype or exclusionary codes and segregating norms. The lack of transparency limits the artist's ability to engage in creative ventures. Discriminatory patterns can also arise when AI tools prioritize certain styles, narratives of culture or demographics over others. The question of disrupting traditional business models and classifying art as a digital asset must also be addressed. Artists must update themselves to technological advancements and acquire novel skills, thereby developing strategies to accelerate the monetization of their work. Also, more inclusive training datasets should be developed, and the implementation of guidelines for ethical AI use in creative domains can be involved.

## 4 Scope for further research

The article is primarily a commentary article that mainly focuses on the emergence of artrepreneurship by integrating Artificial Intelligence. Further research can be conducted on the ethical implications of AI-Art in dealing with ownership and copyrights, originality, bias, and discrimination. Another possible research area is the impact of AI on the art market from both the artist and user perspective, which can provide insights into the valuation and pricing of AI-generated art and the adaptability of art galleries and auction houses. Virtual art as an asset is another area of potential research. Future research can explore the scope of NFT and Blockchain art-driven economies. The social and cultural impact of AI art can also be studied to know the public perceptions. By exploring these domains, a comprehensive and deeper understanding of the intricate relationship between art and AI can be attained.
